# Longitudinal Skeletal and Dental Changes in Untreated Children with Angle Class I and III Malocclusions: A Retrospective Cephalometric Cohort Study

**DOI:** 10.3390/jcm14228037

**Published:** 2025-11-13

**Authors:** Joud A. S. Aljabr, Nabeel Almotairy

**Affiliations:** 1 Cosma Dental Polyclinics, Riyadh 12254, Saudi Arabia; 2 Department of Orthodontics and Pediatric Dentistry, College of Dentistry, Qassim University, Buraidah 52571, Saudi Arabia

**Keywords:** skull/growth and development, maxillofacial development, malocclusion, child, adolescent, cephalometry

## Abstract

**Background:** Class III malocclusion presents complex craniofacial growth patterns, yet longitudinal evidence remains limited compared with Class I malocclusion. This study compared skeletal and dental changes in children with untreated Angle Class I and Class III malocclusions. **Methods:** Forty-eight untreated children (24 Angle Class I and 24 Angle Class III) from the AAOF Craniofacial Growth Legacy Collections were included. Lateral cephalograms were taken at ages 4–5 (T0), 7–8 (T1), 10–11 (T2), and 13–14 (T3). Because the radiographs originated from heterogeneous mid-20th-century X-ray equipment with unknown magnification factors, only angular measurements were used. Cephalometric tracing was conducted using WebCeph™ software with good-to-excellent intra-examiner reliability (κ = 0.71–0.98). Growth changes were assessed using three-way repeated-measures ANOVA, with effect sizes (ηp^2^), mean differences (MDs), and 95% confidence intervals (95% CI) calculated. **Results:** Significant differences in growth patterns were observed between the groups. Class III children showed greater FMA (MD = 6.0°, 95% CI [2.3, 9.7], *p* < 0.05) and gonial angles (MD = 8.1°, 95% CI [3.4, 12.8], *p* < 0.01) at T3, alongside a progressive decrease in ANB (MD = −2.6°, 95% CI [−5.5, −0.3], *p* < 0.01) and A–B mandibular angles (MD = −9.5°, 95% CI [−13.1, −5.9], *p* < 0.05). Class III children also exhibited significant upper incisor proclination and lower incisor retroclination (*p* < 0.001) compared to Class I children. No sex-related differences were observed, except for an SNA angle increase among Class I males (*p* < 0.05). **Conclusions:** Distinct craniofacial growth trajectories were observed between malocclusion classes, with Class III children showing progressive sagittal and vertical divergence from normal growth. The results highlight the need for early detection and monitoring of those at risk for pronounced Class III patterns.

## 1. Introduction

Malocclusion is one of the most prevalent dental conditions worldwide, with significant implications for facial aesthetics, oral function, and psychosocial well-being [1,2]. Among the classifications of malocclusion, Class I and III represent fundamentally different developmental trajectories that manifest through distinct skeletal and dental characteristics. Class I malocclusion, characterised by normal anteroposterior molar relationships with potential crowding or spacing issues, represents the most common classification and serves as the baseline for comparative studies [3]. In contrast, Class III malocclusion presents with a complex three-dimensional facial skeletal imbalance involving a deficient maxillary and/or excessive mandibular development, representing a growth-related dentofacial deformity with a multifactorial aetiology [4]. The prevalence of Class III malocclusion varies significantly among different populations, with higher rates observed in Asian populations compared to Caucasians [5,6].

Skeletal Class III malocclusion is one of the most challenging conditions in clinical orthodontics. Although interest in the treatment of Class III malocclusion has grown in recent years, research on craniofacial growth in untreated Class III individuals remains scarce [7,8]. Although several classic and contemporary studies have examined longitudinal craniofacial growth in Class III malocclusion [9,10,11,12,13,14,15,16,17], most have analysed Class III samples in isolation or compared them to normative growth standards rather than to concurrently observed Class I controls. Moreover, few investigations have traced both Class I and Class III individuals within the same longitudinal dataset, using identical angular variables across successive growth phases. Therefore, evidence directly contrasting the skeletal and dental developmental trajectories of these two malocclusion classes remains limited.

The American Association of Orthodontists Foundation (AAOF) Craniofacial Growth Legacy Collection Project (https://www.aaoflegacycollection.org/) consolidates data from nine major longitudinal studies conducted across the United States and Canada between the 1930s and 1980s, focusing on untreated children and adolescents. The collection contains over 18,900 radiographs and records from 842 subjects, including lateral cephalograms. These records are irreplaceable and cannot be repeated, as ethical considerations nowadays prevent the collection of radiographic information from untreated subjects due to the potential harmful effects of ionising radiation.

Recent advances in longitudinal data modelling, such as mixed-effects growth models, nonlinear regression, and 3D geometric morphometric analyses, have enhanced the understanding of individual growth trajectories and shape variation over time [18,19,20]. However, the application of these advanced statistical or 3D imaging techniques remains limited in historical datasets such as the AAOF Legacy Collection, where only two-dimensional cephalometric data are available. Accordingly, the present study adopts a repeated-measures approach to extract longitudinal information from these irreplaceable records, thereby complementing contemporary modelling frameworks with legacy-based evidence on developmental patterns.

The primary objective of comparative developmental studies between Class I and III malocclusions is to characterise the distinct growth patterns and cephalometric changes that occur throughout the developmental period. Understanding the comparative developmental changes between these two malocclusions is essential for orthodontic diagnosis, treatment planning, and prediction of growth outcomes [21,22,23]. Thus, this study aims to identify, in a longitudinal sample, specific morphological characteristics and growth trajectories that differentiate untreated Angle Class III children from those with untreated Angle Class I occlusion, providing essential baseline data for clinical decision-making.

## 2. Materials and Methods

### 2.1. Study Sample

The patients included in this retrospective cohort study were acquired from the AAOF Legacy Collection. A convenience sampling was employed, where eligible patients should have (1) no prior orthodontic treatment, (2) no congenital craniofacial anomalies, (3) a good-quality lateral cephalogram with full soft tissue display, and (4) radiographic records taken at ages 4–5 years (T0), 7–8 years (T1), 10–11 years (T2), and 13–14 years (T3). The original method used in the AAOF collection to classify patients’ malocclusion was based on clinical examination of molar relationships. However, it was not specified whether this classification was made at baseline (T0) or retrospectively based on later age assessments. Thus, the patients included in the current study were classified into Angle Class I (Class I) or Angle Class III (Class III) malocclusions.

### 2.2. Cephalometric Measurements

**Figure 1:**
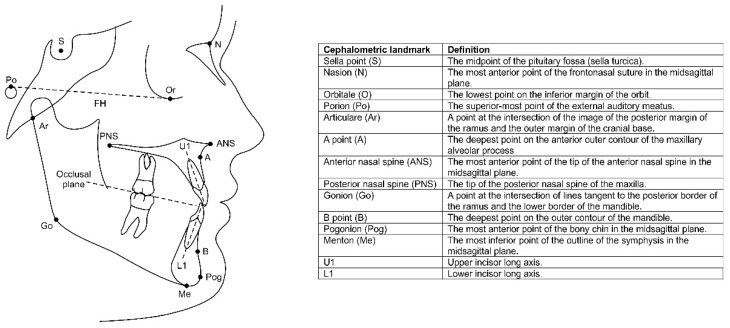
Cephalometric landmarks used in the current study.

The cephalometric assessment was conducted using the WebCeph^TM^ digital cephalometric software (version 2.0.0; AssembleCircle Corp., Hwaseong-si, Republic of Korea). This software is an AI-driven platform for automated cephalometric analysis in orthodontics, previously validated and showing good to excellent agreement with conventional manual methods across a broad range of cephalometric landmarks [24]. Because the radiographs originated from multiple historical AAOF cohorts that used different X-ray machines with unknown and non-standardised magnification factors, only angular measurements were analysed. This restriction ensured internal consistency and minimised potential bias from inter-institutional differences in imaging scale or projection geometry. A total of 20 angles were used to quantify skeletal and dental changes from T0 to T3 (Figure 1; Table 1). The cephalometric tracing was conducted by a single researcher (J.A.S.A.) following training by the senior researcher (N.A.).

Tracing calibration was conducted on 20 randomly selected lateral cephalograms, equally representing the malocclusion and four age groups, acquired from 3 Class I and 3 Class III patients who were not included in the final sample. Two calibration sessions were conducted with a three-week wash-out period. The Weighted Kappa results were good to excellent, ranging from 0.71 (95% CI, 0.41–1) for ANB angle to 0.98 (95% CI, 0.98 to 0.99) for IMPA. The detailed calibration results are presented in Supplementary Material S1. The identities of the calibration and study samples were anonymised with unique codes accessible only to the senior researcher.

### 2.3. Sample Size Calculation

The sample size for the current study was a priori determined using G*Power software (version 3.1.9.6; Heinrich Heine University Düsseldorf, Düsseldorf, Germany). The calculation was for an F test for the within–between interaction in a repeated-measures ANOVA assuming two groups and four timepoints, an effect size f = 0.20, α = 0.05, power (1 − β) = 0.90, a correlation among repeated measures of 0.50, and nonsphericity correction ε = 1.0; the estimated minimum total sample was 46 participants.

### 2.4. Statistics

Statistical analysis was conducted using STATISTICA software (version 12; StatSoft Inc., Tulsa, OK, USA). The mean difference with 95% confidence interval between Class III and Class I malocclusion groups was calculated for each cephalometric variable. Data normality was verified using the Shapiro–Wilk test and Q-Q and histogram plots. The assumption of sphericity for the within-subject factor (time) was verified using Mauchly’s test of sphericity for each angular variable. When the assumption was violated (*p* < 0.05), the Greenhouse-Geisser correction was automatically applied to adjust the degrees of freedom and maintain the validity of the F-statistics. Three-way repeated-measures ANOVA was conducted, where the first factor was the malocclusion class (2 levels; Class I or Class III malocclusions), the second factor was the patient’s sex (2 levels; males or females), and the third factor was each individual cephalometric angle measured at four timepoints (4 levels; T0–T3). The test was conducted to investigate the changes in cephalometric angles within two interactions: (1) Malocclusion class × time, and (2) Malocclusion class × sex × time. Significant main effects were subjected to Tukey’s HSD post hoc test for multiple comparisons. Partial eta squared (ηp^2^) effect size was also calculated. A *p*-value of less than 0.05 was considered statistically significant. We also analysed how key cephalometric angles changed over development from T0 to T3 in the two malocclusion groups (relative change), using measurements at T0 as the baseline from which all subsequent changes (at T1, T2, and T3) were calculated as percentages.

## 3. Results

A total of 48 patients were included, with comparable age and sex distributions between Class I and Class III groups (Table 2). Independent t-tests revealed no significant age differences at T1 and T3 (*p* > 0.05), except at T0 and T2, where Class II children, on average, were older than Class I children. The sample was derived from the Burlington (n = 28), Bolton-Brush (n = 5), Forsyth Twin (n = 5), Fels (n = 3), Oregon (n = 3), Iowa (n = 2), Michigan (n = 1), and Denver (n = 1) growth studies. Participants from the Burlington, Iowa, Denver, and Oregon cohorts were Caucasian, while the remaining cohorts were predominantly Caucasian, though specific information was unavailable.

Descriptive statistics for all cephalometric variables are presented in Table A1, and the Class III–Class I mean differences with 95% confidence intervals are presented in Table 3. No significant developmental or interaction effects were found for SNB, Björk’s sum, APDI, ODI, combination factor, FH–AB, U1–FH, U1–SN, U1–NA, L1–NB, or interincisal angles.

The SNA angle showed a significant class × sex × time interaction (F_3,132_ = 2.86, η_p_^2^ = 0.11, *p* = 0.04; Table A2), with Class I males exhibiting higher SNA at T2–T3 than at T0 (~4% increase, *p* = 0.023 and *p* = 0.026, respectively; Table 4). No significant group differences were observed.

**Figure 2:**
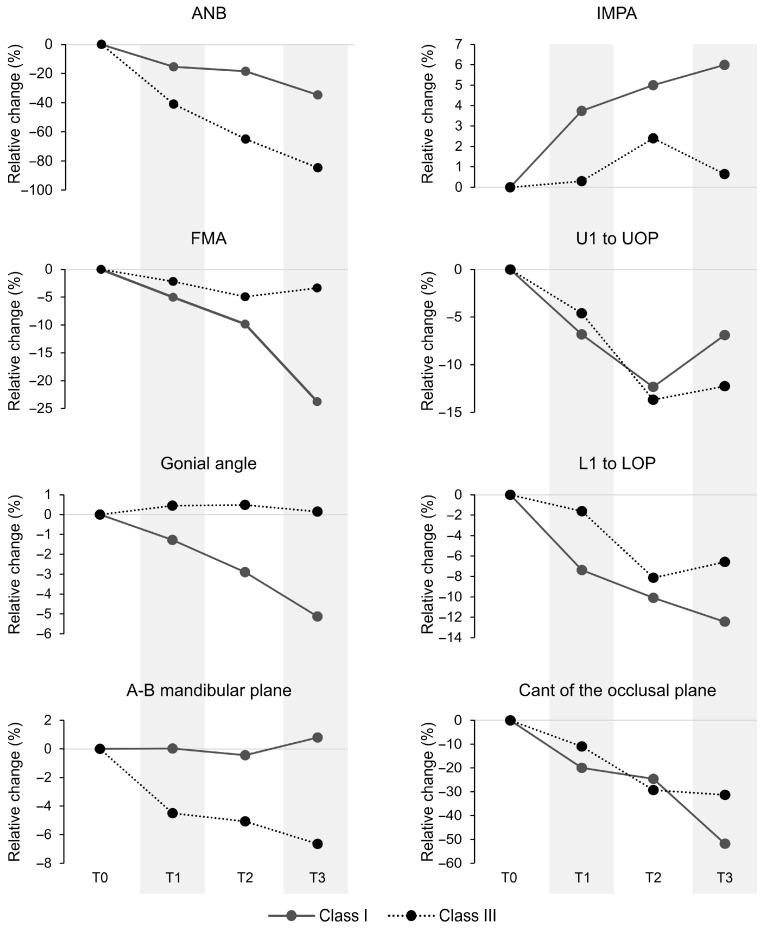
The relative change in significant cephalometric variables at T1–T3 relative to baseline (T0) for Class I and III malocclusion groups.

The ANB angle showed a significant malocclusion class × time interaction (F_3,132_ = 4.64, η_p_^2^ = 0.17, *p* = 0.004). Group comparisons showed that Class I had significantly higher ANB angles at T0 than Class III at T1–T3 (*p* < 0.05), at T1 and T2 than Class III at T2 and T3 (*p* < 0.01), and at T3 than Class III at T3 (*p* = 0.007; Figure 2 and Table 4).

The FMA angle showed a significant malocclusion class × time interaction (F_3,132_ = 7.48, η_p_^2^ = 0.25, *p* < 0.001). Between groups, the Class I angle at T3 was significantly lower than the Class III angles at all time points (*p* < 0.05; Figure 2 and Table 4).

The gonial angle showed a significant malocclusion class × time interaction (F_3,132_ = 4.66, η_p_^2^ = 0.17, *p* = 0.004). Between groups, the Class I gonial angle at T3 was significantly lower than Class III angles at all time points (*p* < 0.01; Figure 2 and Table 4).

The A-B mandibular angle showed a significant malocclusion class × time interaction (F_3,132_ = 4.86, η_p_^2^ = 0.17, *p* = 0.003). Between-group comparisons revealed that Class I angles at T0–T2 were significantly higher than Class III angles at T1–T3, and the Class I angle at T3 was significantly higher than all time points for Class III (*p* < 0.05; Figure 2 and Table 4).

The U1 to UOP angle showed a significant malocclusion class × time interaction (F_3,132_ = 2.68, η_p_^2^ = 0.10, *p* = 0.049). Between groups, the Class I angle at T0 was significantly higher than Class III at T2 and T3 (*p* ≤ 0.01; Figure 2 and Table 4). Additionally, Class I angles at T1–T3 were significantly lower than Class III at T0, and the Class I angle at T2 was lower than Class III at T1 (*p* < 0.001).

The L1 to LOP angle showed a significant malocclusion class × time interaction (F_3,132_ = 3.65, η_p_^2^ = 0.14, *p* = 0.014). Group comparisons showed Class I had significantly lower L1 to LOP at T1 than Class III at T1 and T2 (*p* ≤ 0.01), and Class I angles at T2 and T3 were significantly higher than Class III across all time points (*p* < 0.05; Figure 2 and Table 4).

The IMPA angle showed a significant malocclusion class × time interaction (F_3,132_ = 2.77, η_p_^2^ = 0.11, *p* = 0.044). Group comparisons showed Class I at T0 had significantly higher IMPA than Class III at T0, T1, and T3 (*p* < 0.05), and Class I IMPA at T1–T3 were significantly higher than Class III across all time points (*p* < 0.001; Figure 2 and Table 4).

The cant of the occlusal plane angle showed a significant malocclusion class × time interaction (F_3,132_ = 4.94, η_p_^2^ = 0.18, *p* = 0.003). Between groups, Class I at T0 was higher than Class III at T1–T3 (*p* < 0.05), while Class I at T3 was lower than Class III at T0 and T1 (*p* < 0.01; Figure 2 and Table 4).

## 4. Discussion

This study evaluated longitudinal changes in angular cephalometric variables in children with Angle Class I and Class III malocclusions, highlighting both within-group changes and between-group differences. These findings contribute to understanding dento-skeletal growth dynamics during mixed and early permanent dentition phases, highlighting the importance of early diagnosis, monitoring, and risk assessment. Clinicians should be aware that Class III growth tends to progress unfavourably during childhood and early adolescence, emphasising the value of timely identification of such developmental trends.

Sex-related differences in craniofacial angular measurements were minimal in both Class I and Class III groups, consistent with previous reports [9,10,25,26,27]. The only significant interaction for the SNA angle occurred in Class I males, showing a modest (~4%) increase between ages 10 and 14, likely reflecting anterior maxillary displacement within the normal range of growth variation. Although developmental and genetic influences could not be directly assessed, prior research has attributed sexual dimorphism in craniofacial growth to prolonged male growth duration and later pubertal onset [10,26], as well as genotype-related effects on maxillary projection [28]. However, these explanations should be interpreted as contextually relevant and hypothesis-generating rather than as inferential evidence from the present study.

The SNB angle increased with age in both groups, indicating forward mandibular growth. In contrast, the ANB angle declined significantly from ages 7 to 14, with markedly lower values in Class III (≈85% smaller by age 14). This progressive ANB reduction likely reflects excessive mandibular growth, insufficient maxillary advancement, or both [9,10,17,25,29,30]. The magnitude of decline (≈3.7°) indicates true skeletal divergence rather than positional dental change, aligning with prior findings that anterior and inferior condylar translation drives mandibular prognathism during adolescence [31]. These altered growth patterns may compromise jaw alignment, facial aesthetics, and function. In contrast, the modest ANB decrease in Class I reflects coordinated sagittal advancement of both jaws, preserving balanced relationships [32].

At younger ages (4–5 years), the mean ANB values for Class III children approximated those of Class I, consistent with earlier longitudinal findings that show skeletal divergence typically becomes evident between ages 7 and 10, as mandibular growth accelerates [12]. Hence, early similarity does not imply misclassification of the AAOF cohorts but reflects the expected developmental overlap. It should also be recognised that ANB is sensitive to vertical facial differences, particularly when FMA and gonial angles vary between groups, as observed in the present study [33]. Accordingly, part of the ANB reduction may stem from vertical rotation rather than purely sagittal displacement.

Vertical parameters exhibited distinct developmental trajectories. In Class I, both FMA and gonial angles decreased significantly (~24% and 5%, respectively) by age 13–14, indicating forward mandibular rotation consistent with prior observations in untreated Class I children [10,25,26,34]. In contrast, these angles remained unchanged in Class III, resulting in higher values and reflecting a more vertical growth direction with increased lower facial height [10,13,14,26,35,36]. The A-B mandibular angle remained stable in Class I but decreased by ~6.6% in Class III, indicating a worsening sagittal discrepancy due to disproportionate mandibular or deficient maxillary growth [36].

The occlusal plane angle decreased in both groups, more prominently in Class I (−51.8%) than in Class III (−31.3%) by age 13–14. This likely reflects the combined influence of posterior eruption and forward mandibular rotation in Class I [25,34,37,38]. In contrast, the steeper occlusal plane in Class III indicates clockwise mandibular rotation associated with skeletal imbalance [12,39].

Dental angular changes were modest and compensatory. Both interincisal and U1-SN angles remained stable [26,34], while the U1-UOP decreased from ages 7 to 14, more so in Class III (−12.2%), indicating dentoalveolar adaptation [35]. The L1-LOP also decreased, with greater retroclination in Class III [35]. The IMPA increased (~6%) in Class I by age 13–14 but remained unchanged in Class III, consistent with mandibular morphological constraints [14,26,36]. Such dental adaptations reflect the oral environment’s capacity to respond to aberrant skeletal relationships, but they also highlight the limits of natural compensation [40,41]. Without early identification and intervention, these children are at greater risk for worsening facial disproportions and functional issues as growth continues.

The present findings provide several practical implications for clinicians managing growing patients with developing Class III features. The early and progressive ANB decline, coupled with persistently high FMA and gonial angles, indicates that a skeletal Class III tendency can often be recognised before the late mixed dentition stage (≈7–8 years). Clinically, such patients may initially present with an edge-to-edge incisor relationship or mild anterior crossbite. Detecting this early pattern should prompt periodic cephalometric monitoring rather than deferred observation, as our data demonstrate that divergence from normal growth becomes pronounced by age 10. Moreover, the concurrent sagittal and vertical divergence in Class III implies that treatment planning should extend beyond anteroposterior correction [4]. When vertical parameters remain elevated, clinicians should anticipate potential instability of protraction or camouflage approaches, supporting combined orthopaedic strategies, such as facemask therapy with vertical control or chin-cup components, in suitable cases [42].

For borderline patients, longitudinal monitoring of ANB and A-B mandibular angles can help distinguish transient dental shifts from true skeletal discrepancies. Persistent or worsening angular reductions between the ages of 7 and 10 identify individuals at high risk for progressive skeletal disharmony. In these cases, early interceptive orthopaedic intervention aimed at maxillary advancement and vertical control may improve prognosis and lessen the need for surgical correction, whereas patients maintaining stable profiles may be observed with scheduled reassessment [43].

Due to the limited sample size, Class III children were analysed in the current study as a single diagnostic group, ensuring adequate statistical power but potentially masking subtype-specific trajectories (maxillary hypoplasia, mandibular prognathism, or both). Future longitudinal studies incorporating 3D imaging and larger, demographically balanced samples could clarify whether these subtypes exhibit distinct growth patterns.

Although hierarchical models could theoretically address inter-cohort variability, such analyses were not feasible due to small and uneven subsample sizes across AAOF sub-studies. To evaluate the impact of this limitation, a sensitivity analysis was conducted after excluding the smallest cohorts (<3 participants). The principal results for the ANB (F_3,120_ = 4.48, ηp^2^ = 0.163, *p* = 0.005), FMA (F_3,120_ = 5.59, ηp^2^ = 0.20, *p* = 0.001), and gonial angles (F_3,120_ = 4.44, ηp^2^ = 0.16, *p* = 0.005) remained consistent with the full-sample analyses, suggesting that cohort heterogeneity exerted minimal influence on the overall findings. Nonetheless, future research using larger legacy datasets should explicitly model cohort-level variance.

Beyond corroborating earlier work, this study advances our understanding by directly comparing untreated Class I and Class III trajectories through matched developmental stages. The findings show that skeletal divergence emerges as early as 7–8 years, with Class III children exhibiting sustained mandibular prominence and vertical facial divergence thereafter. These trajectories clarify when and how skeletal imbalances develop, identifying ANB, FMA, and gonial angles as early prognostic indicators for unfavourable Class III progression. Despite limitations inherent to historical data, the standardised longitudinal framework provides internally consistent insight into untreated growth. Collectively, this study offers a valuable benchmark for modern orthodontic assessment by delineating the natural course of Class III development and its clinical implications for timely diagnosis and interceptive management.

### Study Limitations

This study has several limitations. As a retrospective analysis of archived radiographs, it was constrained by the availability and quality of existing records. The AAOF datasets offer a rare longitudinal view of untreated craniofacial growth but represent cohorts from earlier decades under different environmental and technological conditions. Variations in nutrition, health standards, and radiographic equipment since the mid-20th century may influence both growth trajectories and measurement precision. The sample was also predominantly Caucasian, limiting generalizability to the broader population. Therefore, the results should be interpreted as reflecting comparative developmental patterns rather than absolute normative values. Although data were pooled from multiple legacy centres, consistent eligibility criteria and measurement protocols were applied to reduce site-related variation. Future research with larger, more demographically balanced cohorts would benefit from mixed-effects or hierarchical modelling to account for individual and cohort-level variability. Considering potential secular changes in mandibular growth [44] and the influence of genetic and environmental factors, these findings may not fully extend to modern or more diverse populations.

Another methodological limitation arises from the exclusive use of angular variables. Although this choice avoided errors from unknown radiographic magnification, it limits construct validity by omitting linear growth dimensions that are fundamental to craniofacial development. As a result, the observed angular changes reflect directional growth tendencies rather than absolute skeletal expansion and should be interpreted accordingly. Cephalometric analysis itself projects 3D structures onto 2D images and relies on landmark identification, which can introduce tracing errors. The use of a single examiner for cephalometric tracing is a potential source of systematic bias, as inter-examiner variability cannot be assessed. While intra-examiner calibration showed good to excellent reliability for most angles, the wider confidence interval observed for the ANB variable suggests some measurement uncertainty that should be considered when interpreting the results. In this study, each cephalometric variable was analysed separately using a three-way repeated-measures ANOVA across four time points, rather than performing a single multivariate analysis encompassing all angular variables. This approach was chosen to isolate the developmental trajectory of each parameter. However, performing multiple within-variable analyses inherently increases the number of statistical tests and, in turn, the experiment-wise error rate. Although Tukey’s HSD post hoc correction was applied within each variable to reduce Type I error, no additional adjustment (e.g., Holm or Benjamini–Hochberg) was implemented across variables. The results should therefore be interpreted as exploratory and descriptive of individual growth patterns rather than confirmatory across the complete set of variables.

## 5. Conclusions

This study highlighted key developmental differences between Angle Class I and Class III malocclusions from childhood to early adolescence. The developmental trajectories observed in Class I and Class III children differed in both sagittal and vertical dimensions, with Class III children showing a continued divergence from normative craniofacial relationships with growth. Recognising these differing growth trajectories is essential for early identification and monitoring of patients at risk of developing pronounced Class III patterns.

## Figures and Tables

**Table 1:** Cephalometric angular variables used in the current study.

No.	Cephalometric Variable	Definition
1	SNA	Angle between Sella-Nasion-A point.
2	SNB	Angle between Sella-Nasion-B point.
3	ANB	The difference between SNA and SNB.
4	FMA	Angle between Frankfort horizontal plane (FH) and the lower border of the mandible.
5	Gonial angle (Ar-Go-Me)	Angle formed by the posterior border of the ramus and the lower border of the mandible.
6	A-B mandibular angle	Angle between points A and B relative to the lower border of the mandible.
7	Björk’s sum	Sum of saddle (Ar-S-N), articulare (S-Ar-Go), and gonial (Ar-Go-Me) angles.
8	Antero-posterior Dysplasia Indicator (APDI)	Sum of facial angle (FH to NPog) ± A-B plane angle (AB to NPog) ± palatal plane angle (ANS-PNS to FH).
9	Overbite Depth Indicator (ODI)	The arithmetic sum of the A-B mandibular angle and the palatal plane to the Frankfort horizontal plane angle.
10	Combination factor	The sum of APDI and ODI.
11	FH to AB	The angle between Frankfort’s horizontal plane and the AB line.
12	U1 to FH	The angle between the upper incisor long axis (U1) and the Frankfort horizontal plane.
13	U1 to SN	The angle between the upper incisor long axis and the S-N line.
14	U1 to NA	The angle between the upper incisor long axis and the N-A point line.
15	U1 to UOP	Angle between the upper incisor long axis and the occlusal plane.
16	L1 to NB	The angle between the lower incisor long axis (L1) and the N-B point line.
17	L1 to LOP	The angle between the lower incisor long axis and the occlusal plane.
18	IMPA	The angle formed between the lower incisor long axis and the lower border of the mandible.
19	Cant of the occlusal plane	Angle of occlusal plane relative to Frankfort horizontal plane.
20	Interincisal angle	Angle between the upper and lower incisor long axes.

**Table 2:** Number and age distribution of the study sample.

Group		n	T0	T1	T2	T3
			$\bar{x}$ (SD)	$\bar{x}$ (SD)	$\bar{x}$ (SD)	$\bar{x}$ (SD)
Class I	Female	12	4.23 (0.14)	7.3 (0.6)	10.13 (0.28)	13.15 (0.27)
	Male	12	4.19 (0.13)	6.97 (0.49)	10.05 (0.07)	13.05 (0.09)
	Total	24	4.21 (0.13)	7.13 (0.56)	10.09 (0.2)	13.1 (0.21)
Class III	Female	12	4.8 (0.77)	7.45 (0.54)	10.34 (0.47)	13.2 (0.74)
	Male	12	4.65 (0.47)	7.12 (0.3)	10.3 (0.45)	13.12 (0.3)
	Total	24	4.73 (0.64)	7.3 (0.47)	10.32 (0.45)	13.16 (0.56)
*p*-value *	Female		0.02	0.49	0.18	0.82
	Male		0.004	0.4	0.07	0.46
	Total		0.001	0.27	0.03	0.61

n = number; $\bar{x}$ = mean; SD = standard deviation. * Independent *t*-tests.

**Table 3:** Class III–Class I mean difference (MD) with 95% confidence interval (95% CI) for the cephalometric angles used in the current study.

Variable	T0		T1		T2		T3	
	MD	95% CI	MD	95% CI	MD	95% CI	MD	95% CI
SNA	2.38	[0.087, 4.667]	0.77	[−2.101, 3.645]	0.22	[−2.646, 3.088]	0.64	[−2.044, 3.317]
SNB	3.4	[0.608, 6.191]	2.41	[−0.24, 5.051]	2.76	[0.184, 5.337]	3.25	[0.817, 5.675]
ANB	−0.61	[−3.629, 2.418]	−1.63	[−4.313, 1.047]	−2.54	[−4.94, −0.139]	−2.61	[−5.523, 0.305]
Björk’s sum	8.3	[−8.451, 25.048]	2.27	[−0.765, 5.297]	2.71	[−0.418, 5.832]	5.63	[1.762, 9.506]
FMA	0.32	[−2.919, 3.556]	1.1	[−2.333, 4.539]	1.67	[−1.341, 4.682]	6	[2.262, 9.732]
Gonial angle	1.39	[−3.155, 5.939]	3.6	[−0.815, 8.007]	5.7	[1.824, 9.582]	8.1	[3.425, 12.783]
APDI	2.86	[−0.558, 6.284]	6.05	[2.916, 9.19]	5.7	[2.3, 9.103]	4.61	[1.219, 8.003]
ODI	−4.47	[−8.982, 0.046]	−7.49	[−11.017, −3.961]	−7.39	[−10.861, −3.91]	−8.34	[−12.135, −4.54]
Combination factor	−1.27	[−6.456, 3.913]	−1.44	[−5.65, 2.778]	−1.68	[−5.417, 2.05]	−3.73	[−7.864, 0.412]
FH to AB	3.72	[0.325, 7.107]	6.22	[2.985, 9.454]	5.73	[2.606, 8.86]	3.89	[0.272, 7.514]
A-B mandibular plane	−4.03	[−7.643, −0.423]	−7.32	[−10.271, −4.373]	−7.38	[−10.703, −4.048]	−9.47	[−13.061, −5.885]
U1 to FH	0.73	[−4.064, 5.514]	−2.02	[−6.68, 2.643]	1.95	[−2.277, 6.173]	1.41	[−2.779, 5.599]
U1 to SN	0.96	[−4.016, 5.94]	−3.19	[−7.838, 1.451]	0.95	[−3.424, 5.33]	1.8	[−2.635, 6.236]
U1 to UOP	2.02	[−2.284, 6.327]	3.37	[−0.319, 7.05]	0.88	[−2.083, 3.834]	−1.69	[−5.057, 1.674]
IMPA	−6.04	[−10.067, −2.023]	−9.2	[−13.163, −5.244]	−8.56	[−11.709, −5.412]	−10.97	[−14.85, −7.082]
L1 to LOP	4.21	[0.598, 7.825]	8.52	[4.954, 12.095]	5.37	[1.749, 8.989]	8.39	[4.447, 12.331]
Interincisal angle	5.14	[−0.296, 10.576]	10.1	[4.838, 15.369]	4.94	[0.393, 9.493]	3.56	[−1.254, 8.372]
Cant of the occlusal plane	−2.15	[−4.806, 0.513]	−0.52	[−3.621, 2.584]	−2.25	[−5.145, 0.647]	1.7	[−1.074, 4.478]
U1 to NA	−1.44	[−6.94, 4.068]	−3.95	[−8.186, 0.283]	0.73	[−3.023, 4.478]	1.14	[−2.842, 5.119]
L1 to NB	−3.1	[−6.904, 0.708]	−4.52	[−8.342, −0.692]	−3.51	[−6.911, −0.105]	−3.38	[−6.776, 0.006]

**Table 4:** Detailed summary of significant changes in the selected cephalometric angles for Class I and Class III malocclusion groups.

Variable	Significant Interaction	Within-Group Differences		Between-Group Differences
		Class I	Class III	
SNA	Class × sex × time	↑ at T2 & T3 in males (~4%)	N.D.	N.D.
ANB	Class × time	↓ at T3 (~35%)	↓ at T1–T3 (~41% to ~85%)	Class I T0 > Class III T1–T3 Class I T1 & T2 > Class III T2 & T3 Class I T3 > Class III T3
FMA	Class × time	↓ at T3 (~24%)	N.D.	Class I T3 < Class III T0–T3
Gonial angle	Class × time	↓ at T3 (~5%)	N.D.	Class I T3 < Class III T0–T3
A-B mandibular angle	Class × time	N.D.	↓ at T1–T3 (−4.5% to −6.6%)	Class I T0–T2 > Class III T1–T3 Class I T3 > Class III T0–T3
U1 to UOP	Class × time	↓ at T1–T3 (~7% to ~12%)	↓ at T2, T3 (~12% to ~14%)	Class I T0 > Class III T2, T3 Class I T1–T3 < Class III T0 Class I T2 < Class III T1
L1 to LOP	Class × time	↓ at T1–T3 (~7% to ~12%)	↓ at T2, T3 (~7% to ~8%)	Class I T1 < Class III T1, T2 Class I T2 & T3 < Class III T0–T3
IMPA	Class × time	↑ at T2, T3 (~5–6%)	N.D.	Class I T0 > Class III T0, T1 & T3 Class I T1–T3 > Class III at T0–T3
Cant of the occlusal plane	Class × time	↓ at T1–T3 (20% to ~52%)	↓ at T2, T3 (~29%, ~31%)	Class I T0 > Class III T1–T3 Class I T3 < Class III at T0 & T1

Arrows (↑/↓) indicate statistically significant increases or decreases from baseline (T0). N.D. = no observed statistically significant changes. Percentages reflect the approximate change magnitude. Between-group differences refer to statistically significant differences in cephalometric variables between Class I and Class III children at specified time points.

## Data Availability

The source data are publicly accessible via the AAOF Legacy Collection, while the processed dataset used in this analysis is available on request from the corresponding author.

## Supplementary Materials

The following supporting information can be downloaded at https://www.mdpi.com/article/10.3390/jcm14228037/s1, Supplementary Materials S1: Statistical results of data calibration.

## Appendix A

**Table A1:** The average ($\bar{x}$) and standard deviation (SD) for the cephalometric angles used in the current study for Class I and Class III malocclusion groups.

Variable		Class I; $\bar{x}$ (SD)				Class III; $\bar{x}$ (SD)			
		T0	T1	T2	T3	T0	T1	T2	T3
SNA	Female	81.91 (3.19)	81.82 (3.41)	81.52 (3.95)	81.86 (2.64)	81.69 (2.88)	81.03 (2.36)	81.39 (3.96)	81.92 (2.5)
	Male	77.83 (4.24)	78.97 (4.59)	81.03 (2.48)	80.99 (2.71)	82.9 (4.43)	81.33 (4.63)	81.63 (5.07)	82.23 (5.26)
	Total	79.87 (4.22)	80.39 (4.21)	81.28 (3.24)	81.42 (2.65)	82.25 (3.64)	81.17 (3.5)	81.5 (4.4)	82.06 (3.91)
SNB	Female	75.1 (3.74)	76.93 (3.05)	77.05 (3.65)	78.19 (3.31)	77.44 (3.32)	78.53 (3.22)	79.61 (3.35)	81.27 (3.28)
	Male	73.76 (3.44)	75.36 (3.82)	77.32 (2.39)	78.09 (2.44)	78.3 (3.55)	78.58 (3.16)	80.34 (3.57)	81.51 (3.28)
	Total	74.43 (3.58)	76.15 (3.47)	77.19 (3.02)	78.14 (2.84)	77.83 (3.38)	78.55 (3.12)	79.95 (3.4)	81.38 (3.21)
ANB	Female	5.98 (2.21)	4.88 (1.76)	4.47 (2.64)	3.67 (1.99)	4.25 (1.8)	2.5 (1.81)	1.77 (3.17)	0.64 (2.22)
	Male	4.07 (2.06)	3.61 (1.97)	3.71 (1.42)	2.9 (1.8)	4.61 (3.33)	2.75 (2.97)	1.29 (3)	0.71 (3.33)
	Total	5.02 (2.31)	4.25 (1.94)	4.09 (2.11)	3.28 (1.9)	4.42 (2.56)	2.61 (2.36)	1.55 (3.04)	0.68 (2.72)
Björk’s sum	Female	395.07 (5.07)	393.4 (4.49)	392.83 (4.87)	390.11 (7.83)	395.58 (4.54)	395.97 (4.52)	395.34 (3.16)	395.54 (5.57)
	Male	379.18 (57.09)	393.29 (4.52)	391.58 (3.45)	388.39 (4.92)	395.24 (3.6)	395.18 (3.88)	394.4 (6.11)	394.1 (5.69)
	Total	387.12 (40.46)	393.34 (4.41)	392.2 (4.18)	389.25 (6.45)	395.42 (4.05)	395.61 (4.17)	394.91 (4.66)	394.88 (5.55)
FMA	Female	27.39 (5.38)	26.91 (6.28)	26.13 (4.68)	22.07 (7.4)	27.81 (3.63)	27.33 (5.1)	26.26 (3.62)	27.4 (5.58)
	Male	28.3 (5.96)	26 (5.45)	24.09 (4.5)	20.39 (4.65)	28.58 (3.01)	27.82 (2.94)	27.4 (4.27)	27.01 (5.23)
	Total	27.85 (5.57)	26.45 (5.77)	25.11 (4.61)	21.23 (6.1)	28.17 (3.31)	27.56 (4.17)	26.78 (3.88)	27.22 (5.31)
Gonial angle	Female	126.86 (8.25)	124.98 (8.78)	124.08 (5.46)	121.25 (10.1)	127.42 (6.83)	129.27 (6.67)	128.04 (6.1)	128.4 (6.97)
	Male	127.57 (8.29)	126.2 (7.23)	122.97 (5.96)	120.14 (6.02)	130.01 (5.45)	129.09 (5.02)	130.64 (6.41)	129.26 (6.57)
	Total	127.21 (8.09)	125.59 (7.89)	123.53 (5.62)	120.69 (8.15)	128.61 (6.25)	129.19 (5.85)	129.23 (6.24)	128.8 (6.65)
APDI	Female	77.13 (3.75)	78.13 (3.49)	79.76 (6.35)	81.32 (5.44)	79.83 (4.37)	83.54 (5.7)	84.2 (5.16)	84.64 (5.07)
	Male	78.01 (5.54)	78.16 (3.88)	78.6 (2.55)	80.43 (3.23)	81.15 (6.91)	84.98 (5.19)	85.68 (5.36)	86.48 (6.49)
	Total	77.57 (4.65)	78.15 (3.61)	79.18 (4.77)	80.87 (4.4)	80.44 (5.58)	84.2 (5.4)	84.88 (5.19)	85.48 (5.71)
ODI	Female	78.24 (6.26)	78.86 (5.48)	78.02 (5.56)	77.49 (7.36)	73.08 (5.54)	69.2 (4.97)	69.73 (5.75)	67.83 (5.72)
	Male	78.43 (10.29)	77.11 (4.89)	75.86 (4.47)	75.83 (4.25)	74.8 (5.83)	72.03 (5.8)	69.36 (5.18)	68.91 (5.88)
	Total	78.34 (8.33)	77.98 (5.16)	76.94 (5.06)	76.66 (5.94)	73.87 (5.62)	70.49 (5.44)	69.56 (5.38)	68.33 (5.69)
Combination factor	Female	154.71 (8.21)	156.99 (6.55)	157.78 (6.92)	158.81 (7.94)	152.92 (6.06)	152.74 (7.03)	153.92 (6.16)	152.48 (6.45)
	Male	156.45 (12.93)	155.27 (6.09)	154.46 (4.11)	156.26 (4.84)	155.95 (2.95)	157 (6.51)	155.04 (5.11)	155.38 (6.42)
	Total	155.58 (10.62)	156.13 (6.25)	156.12 (5.82)	157.54 (6.56)	154.3 (5.03)	154.69 (7)	154.44 (5.61)	153.81 (6.46)
FH to AB	Female	75.33 (4.41)	76.18 (4.03)	77.8 (4.78)	80.05 (6.88)	79.47 (3.92)	83.51 (5.59)	84.1 (5.06)	84.7 (5.52)
	Male	75.64 (5.42)	77.53 (4.69)	79.32 (3.95)	82.1 (3.69)	78.89 (6.41)	82.56 (4.71)	84.51 (4.69)	85.28 (5.92)
	Total	75.49 (4.83)	76.85 (4.33)	78.56 (4.36)	81.07 (5.5)	79.2 (5.1)	83.07 (5.12)	84.29 (4.79)	84.97 (5.59)
A-B mandibular plane	Female	77.28 (4.34)	76.91 (4.93)	76.07 (5.94)	77.06 (7.16)	72.72 (4.75)	69.16 (3.79)	69.63 (4.65)	67.89 (4.77)
	Male	76.06 (6.45)	76.48 (3.65)	76.59 (3.55)	77.51 (3.74)	72.53 (6.39)	69.61 (4.62)	68.15 (5.41)	67.71 (5.88)
	Total	76.67 (5.41)	76.69 (4.25)	76.33 (4.79)	77.28 (5.59)	72.63 (5.43)	69.37 (4.1)	68.95 (4.96)	67.81 (5.19)
U1 to FH	Female	99.69 (6.49)	107.53 (8.01)	108.36 (7.76)	109.02 (7.83)	100.2 (8.51)	104.8 (7.04)	112.11 (6.01)	110.08 (6.7)
	Male	98.85 (9.63)	105.29 (8.78)	110.44 (6.81)	110.24 (5.56)	99.75 (5.96)	103.91 (5.87)	110.45 (6.2)	112.16 (6.42)
	Total	99.27 (8.04)	106.41 (8.3)	109.4 (7.22)	109.63 (6.67)	99.99 (7.29)	104.39 (6.41)	111.35 (6.03)	111.04 (6.51)
U1 to SN	Female	92.01 (7.47)	101.04 (7.37)	101.67 (8.74)	100.98 (8.42)	92.44 (8.72)	96.15 (7.03)	103.12 (6.74)	101.94 (7.52)
	Male	91.26 (9.67)	98.02 (9.41)	102.95 (5.7)	102.24 (5.55)	92.78 (6.44)	96.55 (5.38)	103.43 (6.55)	105.14 (6.48)
	Total	91.64 (8.46)	99.53 (8.41)	102.31 (7.25)	101.61 (7.01)	92.6 (7.6)	96.33 (6.2)	103.26 (6.51)	103.41 (7.1)
U1 to UOP	Female	65.31 (6.32)	58.95 (5.47)	57.29 (4.23)	60.46 (4.67)	67.19 (7)	62.17 (4.38)	57.34 (4.7)	59.61 (5.35)
	Male	64.48 (9)	61.99 (6.61)	56.5 (3.48)	60.38 (4.11)	66.59 (4.35)	65.81 (4.84)	58.27 (3.57)	57.68 (5.71)
	Total	64.89 (7.62)	60.47 (6.13)	56.89 (3.81)	60.42 (4.3)	66.91 (5.82)	63.84 (4.86)	57.77 (4.16)	58.73 (5.48)
IMPA	Female	92.65 (5.73)	95.59 (5.77)	95.87 (4.15)	95.72 (5.91)	84.61 (5.98)	85.68 (5.83)	87.28 (3.32)	84.2 (4.44)
	Male	89.78 (7.57)	93.67 (5.46)	95.68 (4.74)	97.65 (5.33)	85.83 (5.65)	85.13 (7.86)	87.14 (6.4)	87.52 (7.78)
	Total	91.22 (6.73)	94.63 (5.58)	95.78 (4.36)	96.68 (5.59)	85.17 (5.74)	85.43 (6.68)	87.22 (4.86)	85.72 (6.28)
L1 to LOP	Female	75.37 (4.77)	69.39 (4.46)	68.21 (5.5)	67.88 (6.17)	80.51 (4.73)	79.55 (3.49)	74.01 (3.84)	75.91 (4.66)
	Male	76.71 (5.52)	71.47 (6.98)	68.51 (5.3)	65.3 (5)	79.95 (7)	78.25 (6.21)	73.4 (7.35)	73.87 (8.42)
	Total	76.04 (5.09)	70.43 (5.83)	68.36 (5.28)	66.59 (5.65)	80.25 (5.75)	78.96 (4.85)	73.73 (5.59)	74.98 (6.57)
Interincisal angle	Female	140.27 (6.34)	129.97 (8.21)	129.63 (8.45)	133.2 (8.15)	147.37 (8.84)	142.2 (8)	134.34 (7.18)	138.31 (5.33)
	Male	143.07 (10.85)	135.08 (10.29)	129.79 (7.69)	131.72 (8.19)	146.15 (9.27)	143.14 (7.31)	135.03 (5.93)	133.31 (8.76)
	Total	141.67 (8.81)	132.52 (9.47)	129.71 (7.9)	132.46 (8.03)	146.81 (8.86)	142.63 (7.54)	134.66 (6.5)	136.02 (7.4)
Cant of the occlusal plane	Female	15.14 (2.94)	12.78 (5.13)	12.47 (4.01)	8.29 (3.98)	12.82 (2.99)	12.81 (4.56)	9.15 (4.32)	9.01 (4.27)
	Male	15.8 (3.8)	11.98 (4.08)	10.87 (4.03)	6.61 (2.81)	13.91 (3.68)	10.74 (4.46)	9.74 (4.19)	9.32 (3.82)
	Total	15.47 (3.34)	12.38 (4.55)	11.67 (4.02)	7.45 (3.47)	13.32 (3.3)	11.86 (4.54)	9.42 (4.18)	9.15 (3.99)
U1 to NA	Female	10.1 (7.87)	19.22 (6.6)	20.15 (6.72)	19.12 (7.21)	10.75 (7.71)	15.13 (5.59)	21.73 (4.48)	20.03 (5.69)
	Male	13.48 (11.91)	19.02 (8.19)	21.92 (5.4)	21.25 (4.74)	9.88 (8.07)	15.22 (6.71)	21.8 (6.63)	22.85 (6.92)
	Total	11.79 (10.02)	19.12 (7.27)	21.03 (6.03)	20.18 (6.07)	10.35 (7.72)	15.17 (5.99)	21.76 (5.44)	21.32 (6.31)
L1 to NB	Female	23.65 (3.38)	25.92 (4.96)	26.06 (4.31)	24.01 (4.21)	17.62 (4.76)	20.18 (4.22)	21.41 (4.07)	19.45 (3.45)
	Male	19.39 (8.09)	22.29 (7.22)	23.75 (6.22)	24.13 (5.56)	19.37 (5.65)	18.9 (6.58)	21.38 (5.84)	22.14 (6.88)
	Total	21.52 (6.44)	24.11 (6.33)	24.9 (5.37)	24.07 (4.82)	18.42 (5.14)	19.59 (5.34)	21.4 (4.84)	20.69 (5.35)

**Table A2:** Within-group statistical differences and T0 to T1–T3 relative changes in cephalometric variables.

The ANB angle in Class I malocclusion was 35% lower at T3 than at T0 (*p* = 0.002; Figure 2). In Class III, it was significantly lower at T1, T2, and T3 compared to T0 (−41%, −65%, and −85%, respectively; *p* < 0.01). The FMA angle in Class I malocclusion was significantly lower at T3 than at T0–T2 (*p* < 0.001), with a 24% reduction from T0 (Figure 2). No significant changes were found in Class III. Similarly, the gonial angle in Class I malocclusion was significantly lower at T3 than at T0 and T1 (*p* < 0.01), with a 5% reduction from T0 (Figure 2). No significant changes were observed for Class III. The A–B mandibular angle in Class I malocclusion showed no significant changes across time points. In contrast, Class III malocclusion showed significant reductions at T1 (−4.5%), T2 (−5.1%), and T3 (−6.6%) compared to T0 (*p* < 0.05; Figure 2). The U1 to UOP angle in Class I malocclusion was significantly higher at T0 than at T1–T3 (*p* < 0.05), with reductions of 6.8% at T1, 12.3% at T2, and 6.8% at T3 (Figure 2). In Class III, the angle at T0 was significantly higher than at T2 and T3 (*p* < 0.001), with reductions of 13.6% and 12.2%, respectively. The L1 to LOP angle in Class I was significantly higher at T0 than at T1–T3 (*p* < 0.001; reductions of 7.4%, 10.1%, and 12.4%, respectively; Figure 2), and T1 was higher than T3 (*p* = 0.014). In Class III, angles at T0 and T1 were significantly higher than at T2 and T3 (*p* < 0.001), with reductions of 8.1% and 6.5% from T0. The IMPA angle in Class I increased significantly at T2 and T3 compared to T0 (by ~5% and 6%, respectively; *p* < 0.05; Figure 2), with no significant changes in Class III. The cant of the occlusal plane angle in Class I was significantly higher at T0 than at T1 (−20%), T2 (−24.6%), and T3 (−51.8%) (*p* < 0.001; Figure 2), with T3 also significantly higher than T1 and T2 (*p* < 0.001). In Class III, T0 was significantly higher than T2 (−29.3%) and T3 (−31.3%) (*p* < 0.001), and T1 was higher than T3 (*p* = 0.023).
